# Targeting JAK-STAT Signalling Alters PsA Synovial Fibroblast Pro-Inflammatory and Metabolic Function

**DOI:** 10.3389/fimmu.2021.672461

**Published:** 2021-06-24

**Authors:** Aisling O’Brien, Megan Mary Hanlon, Viviana Marzaioli, Siobhan C. Wade, Keelin Flynn, Ursula Fearon, Douglas J. Veale

**Affiliations:** ^1^ Molecular Rheumatology, School of Medicine, Trinity Biomedical Sciences Institute, Trinity College Dublin, Dublin, Ireland; ^2^ Rheumatology European League against Rheumatism (EULAR) Centre of Excellence, Centre for Arthritis & Rheumatic Diseases, St Vincent’s University Hospital, University College Dublin, Dublin, Ireland

**Keywords:** psoriatic arthritis, metabolism, JAK-STAT (janus kinase-signal transducer and activators of transcription), synovial fibroblast, synovial invasion

## Abstract

**Objectives:**

Psoriatic arthritis (PsA) is a chronic inflammatory disease associated with psoriasis. Janus Kinase inhibitors (JAKi) have emerged as an encouraging class of drugs for the treatment of PsA. Here, we compare the effect of four JAKi on primary PsA synovial fibroblasts (PsAFLS) activation, metabolic function, and invasive and migratory capacity.

**Methods:**

Primary PsAFLS were isolated and cultured with JAKi (Peficitinib, Filgotinib, Baricitinib and Upadacitinib) in the presence of Oncostatin M (OSM). pSTAT3 expression in response to OSM was quantified by Western Blot analysis. Pro-inflammatory cytokines/chemokines were quantified by ELISA and cell migration by wound-repair scratch assays. Invasive capacity was examined using Matrigel™ invasion chambers and MMP multiplex MSD assays. PsAFLS bioenergetics was assessed using the Seahorse XF^e^ Extracellular Flux Analyzer, which simultaneously quantifies two energetic pathways- glycolysis (ECAR) and oxidative phosphorylation (OCR). In parallel, inflammatory, invasive, and migratory genes were quantified by RT-PCR.

**Results:**

OSM induces pSTAT3 expression in PsAFLS. OSM-induced secretion of MCP-1 and IL-6 was inhibited by all JAKi with Peficitinib, Baricitinib and Upadacitinib showing the greatest effect. In contrast, JAKi had no significant impact on IL-8 expression in response to OSM. PsAFLS cell invasion, migratory capacity and MMP1, 3, and 9 were suppressed following JAKi treatment, with Peficitinib showing the greatest effect. These functional effects were accompanied by a change in the cellular bioenergetic profile of PsAFLS, where JAKi significantly decreased glycolysis and the ECAR/OCR, resulting in a shift to a more quiescent phenotype, with Peficitinib demonstrating the most pronounced effect.

**Conclusion:**

This study demonstrates that JAK/STAT signalling mediates the complex interplay between inflammation and cellular metabolism in PsA pathogenesis. This inhibition shows effective suppression of inflammatory mechanisms that drive pathogenic functions of PsAFLS, further supporting the role of JAKi as a therapeutic target for the treatment of PsA.

## Introduction

Psoriatic arthritis (PsA) is a chronic disease characterised by joint destruction and associated psoriasis (PsO) (1, 2). PsA synovitis is characterised by dysfunctional angiogenesis, followed by infiltration of both innate and adaptive immune cells. This leads to proliferation and activation of synovial fibroblast cells (FLS), a major source of pro-inflammatory mediators and matrix-degrading enzymes which orchestrate the persistent infiltration of immune cells and invade adjacent cartilage and bone (1–4). Furthermore, PsAFLS can further induce angiogenesis, promoting a more dysregulated endothelial cell (EC) phenotype compared to that of rheumatoid arthritis synovial fibroblast-like cells (RAFLS) (5), a phenotype consistent with the macroscopic appearance of blood vessels *in vivo* (6, 7). Indeed, PsAFLS alter the morphology, migratory and adhesive functions of ECs, in addition to their metabolic profile (5).

Many proinflammatory cytokines have been implicated in the pathogenesis of PsA, including TNF, IL-17A and IL-12/IL-23 pathways which play a key role in promoting the inflammatory response (8–11). Recently, targeted agents developed for PsA treatment include inhibitors of the Janus-Kinase (JAK) family of receptor-associated tyrosine kinases (12). Activated JAKs recruit and activate signal transducer and activator of transcription (STATs), which in turn drives gene transcription (13, 14). There are four JAK isoforms: JAK1, JAK2, JAK3 and TYK2, which depending on their stimulus induce the phosphorylation of different STAT proteins. Despite ongoing clinical trials, few studies have examined the precise effect of these agents in PsA synovial tissue cell subtypes, and thus our understanding of the comparative effect by which they reduce inflammation in the PsA joint is limited. Fiocco et al. demonstrated increased expression of the JAK1/STAT3/STAT5 transcriptional network associated with joint specific T cell populations in PsA (15). Consistent with this, studies have demonstrated in PsA that Tofacitinib, a JAKi, can regulate the frequency of pathologic CD4^+^CD11a^+^CD45RO^+^IL-17^+^ T cells (16), inhibit Mo-DC differentiation through NOX5 and ROS production (17), decrease the T cell stimulatory capability of dendritic cells through suppression of type-I-IFN signalling (18), in addition to suppression of enthesitis in a A20^myelKO^ animal model (19). Furthermore, we and others have shown increased expression of STAT signalling components in PsA synovial-biopsies and FLS (19, 20), with tofacitinib inhibiting spontaneous release of pro-inflammatory cytokines from *exvivo* PsA synovial explant cultures, in addition to inhibition of PsAFLS migratory and invasive capacity (21).

Current therapeutic options for PsA are mainly monoclonal antibody drugs targeting TNF, IL-23 or IL-17 (22). The therapeutic responses to these biologic disease modifying anti-rheumatic drugs (bDMARDs) can vary greatly with some patients showing inadequate responses. As the JAK/STAT pathway is central in driving both pro- and anti-inflammatory signals in immune regulation, including pathways which are involved in the pathogenesis of PsA, JAKi are now of great interest as a treatment option for PsA patients (23, 24). Currently, Tofacitinib is the only JAKi approved for PsA, with Peficitinib, Filgotinib, Baricitinib and Upadacitinib in clinical trials or undergoing pre-clinical evaluation (25). Upadacitinib (SELECT- PsA 2) and Filgotinib (PENGUIN 2), both JAK1 inhibitors, are currently in placebo controlled, double-blind phase III trials for the treatment of PsA patients with inadequate responses to at least 1 DMARD (26, 27). In addition, approximately 25% of patients with moderate-severe PsO develop PsA. Peficitinib, a pan JAKi and Baricitinib a JAK1/2 inhibitor have been shown to significantly improve both clinical and histological manifestations of this skin disease in phase II clinical trials (28, 29).

As head-to-head comparisons are difficult to perform, the aim of this study was to directly compare the effect of Peficitinib, Filgotinib, Baricitinib and Upadacitinib on PsAFLS inflammatory responses, migratory and invasive capacity, in addition to their effect on the metabolic profile of these cells.

## Materials and Methods

### Patient Recruitment and Arthroscopy

PsA patients were recruited from the Rheumatology Department, St. Vincent’s University Hospital. Ethics for this study was approved by the St. Vincent’s University Hospital Ethics and Medical Research Committee and was performed in accordance with the Declaration of Helsinki. All patients gave fully informed written consent. PsA patients were defined according to CASPAR criteria. Baseline demographics of the PsA cohort are shown in **Table 1**. Arthroscopies were performed under local anaesthetic using a Wolf 2.7 mm needle, and synovial tissue biopsies were obtained from the site of inflammation under direct visualisation as previously described (6). Biopsies were utilised for isolation of primary PsA synovial fibroblasts (PsAFLS). Ethics approval number RS18-055.

**Table 1:** Baseline Characteristics and clinical features of PsA Patients.

Demographic and Clinical Data	PsA (n = 14)
Female sex n (%)	10 (71.4)
Age (years)	53.8 ± 14.3
Disease duration (Years)	11.7 ± 13.1
ESR (mm/h)	25.9 ± 28.2
CRP (mg/L)	7.5 ± 12.6
No. tender joints	2 ± 1.8
No. swollen joints	1.1 ± 1.2
Pain VAS	57.5 ± 17.8
DAS28	3.1 ± 0.9
DMARDs (in last 3 months)	36%

Data presented as Mean (SD). ESR, erythrocyte sedimentation rate; CRP, C-reactive protein; TJC28, tender joint count out of 28 joints; SJC28, swollen joint count out of 28 joints; VAS, visual analog scale; DAS28, disease activity score based on 28-joint count; DMARD, disease-modifying antirheumatic drugs.

### Isolation of Primary Fibroblasts

PsA synovial biopsies were digested with 1 mg/ml collagenase type 1 (Worthington Biochemical, Freehold, NJ, USA) in RPMI-1640 (Gibco-BRL, Paisley, UK) for 4 h at 37°C in humidified air with 5% CO_2_. Dissociated cells were grown to confluence in RPMI-1640, 10% FBS (Gibco-BRL), 10 ml of 1 mmol/l HEPES (Gibco-BRL), penicillin (100 units/ml; Biosciences), streptomycin (100 units/ml; Biosciences) and fungizone (0.25 μg/ml; Biosciences) before passaging. Cells were used between passages 2–8.

### Stimulation of PsAFLS

PsAFLS were seeded in 6- (1x10^5^ cells/well), 48- (2x10^4^ cells/well) or 96- (2x10^4^ cells/well) well plates and allowed to attach overnight in RPMI-1640, 10% FBS, 10 ml of 1 mmol/l HEPES, penicillin, streptomycin and fungizone. Media was removed the following day and cells were serum starved by adding RPMI-1640 containing 1% FBS, 10 ml of 1 mmol/l HEPES, penicillin, streptomycin and fungizone for a further 24 h. PsAFLS were then pretreated with JAKi (Peficitinib (PEF), Filgotinib (FILGO), Baricitinib (BARI) (ACHEMBLOCK, CA,USA) and Upadacitinib (UPA) (Ambeed Inc, IL, USA); 5 μM) (JAKi were reconstituted in DMSO to 100 mM) or DMSO (5 μM; Sigma Aldrich) (vehicle control) for 1 h before being stimulated with Oncostatin M (OSM) (10 ng/ml; R&D) for 24 h. Concentration of 5 µM for all JAKi was used based on a previous study showing the dose response of all JAKi in FLS (29, 30). Additional experiments were performed to examine if JAKi alter secondary downstream effects of cytokines that do not signal through the JAK-STAT. Therefore, PsAFLS were plated in a 96- (2x10^4^ cells/well) well as outlined above and stimulated with IL-1β (10 ng/ml; Bio-Techne LTD, UK) +/- JAKi, with a DMSO control. Additional experiments were also performed for Tofacitinib (5 µM) under OSM (10 ng/ml) or IL-1β (10 ng/ml) stimulation.

### Protein Isolation and Western Blot Analysis

To determine the effect of OSM on pSTAT3 expression, PsAFLS (1x10^5^ cells/well) were seeded in 6-well plates. Once confluent, cells were serum starved as previously described and stimulated with OSM (10 ng/ml) overnight, unstimulated (basal) PsAFLS were used as a control. Media was removed from the PsAFLS and ice-cold RIPA (Radio-Immunoprecipitation Assay) buffer (Sigma) containing 10 μg/ml phosphatase inhibitor cocktail and 10 μg/ml protease inhibitor cocktail (Sigma) was used to extract protein from the PsAFLS. Measurement of protein concentration was performed using a BCA assay (Pierce Chemical Co, Rockford, IL, USA). Protein (3 μg) was resolved by SDS-PAGE (5% stacking, 10% resolving), resolved proteins were then transferred onto nitrocellulose membranes (Amersham Biosciences, Buckinghamshire, UK) prior to 1 h blocking in wash buffer containing 5% non-fat milk with gentle agitation at room temperature. Membranes were incubated with rabbit polyclonal anti-pSTAT3 (Cell-Signaling Technology, UK), diluted in 5% non-fat milk containing 0.1% Tween 20 at 4°C overnight with gentle agitation. β-actin (1:5000, Sigma) was used as a loading control. Following three 15 min washes, membranes were incubated with appropriate horseradish peroxidase-conjugated secondary antibodies (1:5000) for 3 h at room temperature. The signal was detected using SuperSignal^®^ West Pico Chemiluminescent Substrate (Amersham Biosciences). Band densities were imaged using the ChemiDoc MP Imaging System (Bio-Rad, USA).

### Enzyme-Linked Immunosorbent Assay

Supernatants from treated PsAFLS and DMSO control (5 μM) (2x10^4^ cells/well) seeded in 96-well plates were harvested and levels of IL-8, IL-6, and MCP-1 measured by specific ELISA (MCP-1: eBiosciences, USA, IL-8, IL-6; DuoSet ELISA, R&D systems, UK) according to manufacturer’s protocol.

### mRNA Extraction and cDNA Synthesis

To determine the effects of JAKi on specific genes in response to OSM stimulation, PsAFLS (1x10^5^ cells/well) were seeded in 6-well plates and stimulated as previously described. Total RNA was isolated using an RNeasy Plus mini kit (Qiagen, Germany) according to the manufacturer’s specifications. The integrity of the RNA samples was assessed using a bioanalyzer (Agilent, CA, USA). Samples with a 260:280 nm ratio of 1.8 or above were used in subsequent experiments. Total RNA (100 ng) was reverse transcribed to cDNA using a high capacity cDNA reverse transcription kit (Applied Biosystems, Cheshire, UK) and stored at -20°C until further use.

### RT-PCR Analysis

Gene expression data were quantified by RT-PCR using the QuantStudio 5 Thermal Cycler (Applied Biosystem, Lewes, UK). Reaction mixtures contained 1 μl of cDNA, SYBR green PCR mastermix (Applied Biosystems) and target mRNA specific primer pairs as follows: IL-6 for 5’ CCCTGAGAAAGGAGACATTGTAAC 3’, IL-6 rev 5’CCTCTTTGCTGCTTTCACACATG 3’, IL-8 for 5’ TTGGCAGCCTTCCTGATTTC 3’, IL-8 rev 5’ TGGCAAAACTGCACCTTCAC 3’, MCP-1 for 5’ GCTCGCTCAGCCAGATGCAA 3’, MCP-1 rev 5’ TGGTGAAGTTATAACAGCAGGTGA 3’, MMP1 for 5’ GCTAACAAATACTGGAGGTATGATG 3’, MMP1 rev 5’ ATTTTGGGATAACCTGGATCCATAG 3’, ICAM for 5′ AACCAGAGCCAGGAGACACTG 3′, ICAM rev 5′ GCGCCGGAAAGCTGTAGATG 3′.

Samples lacking multiscribe reverse transcriptase formed the negative controls to ensure target-specific quantification. Data were analysed using the comparative threshold cycle (Ct) method with normalization to the expression of RPLPO (for 5′ GCGTCCTCGTGGAAGTGACATCG 3′, rev 5′ TCAGGGATTGCCACGCAGGG 3′) and HPRT1 (for 5′ ATGGACAGGACTGAACGTCTTG 3′, rev 5′ GGCTACAATGTGATGGCCTC 3′) as endogenous controls.

### Cellular Bioenergetic Function Analysis

Oxygen consumption rate (OCR) and extracellular acidification rate (ECAR), reflecting oxidative phosphorylation and glycolysis, respectively, were measured using the Seahorse-XFe96 analyser (Seahorse Biosciences, UK). PsAFLS were seeded at 12x10^3^/well in a 96-well cell culture XFe microplate (Seahorse Biosciences) and allowed to adhere overnight. Following this, cells were treated with JAKi/DMSO (5 μM) for 1 h and then stimulated with OSM (10 ng/ml) for 24 h. Additional experiments were also performed in the presence of IL-1β (10 ng/ml). Basal oxidative phosphorylation/glycolysis were calculated by the average of five baseline OCR/ECAR measurements, respectively, obtained before injection of specific metabolic inhibitors; oligomycin (ATP-synthase-inhibitor) (2 μg/ml; Seahorse Biosciences), trifluorocarbonylcyanide phenylhydrazone (FCCP) (mitochondrial uncoupler) (5 μM; Seahorse Biosciences) and antimycin A (complex-III inhibitor) (2 μM; Seahorse Biosciences) and rotenone (2 μM; Sigma Aldrich). Oligomycin was injected to evaluate both the maximal glycolytic rate and ATP synthesis, determined by subtracting the amount of respiration left after oligomycin injection from baseline OCR. FCCP was injected to evaluate the maximal respiratory capacity (average of three measurements following injection). Maximal respiratory capacity was determined by subtracting baseline OCR from FCCP-induced OCR and the respiratory reserve (baseline OCR subtracted from maximal respiratory capacity).

### Migration Assay

PsAFLS (2x10^4^ cells/well) were seeded in 48-well plates for 24 h and serum starved as previously described. A single scratch wound was induced through the middle of each well with a sterile pipette tip and cells were subsequently treated with JAKi/DMSO (5 μM) for 1 h followed by stimulation with OSM (10 ng/ml) for 24 h.

PSA FLS migration across the wound margins was assessed and photographed using a phase-contrast microscope. Semi-quantitative analysis of cell repopulation of the wound was assessed. Briefly, cells were fixed with 4% paraformaldehyde, stained with 0.1% crystal violet and the number of migrating cells across the time zero margin was assessed.

### Transwell Invasion Assay

BioCoat Matrigel™ Invasion Chambers (Becton Dickinson, UK) were used to assess PsAFLS invasion. Cells were seeded at 3x10^4^ cells/well in the migration chamber on 8 μM membranes pre-coated with matrigel. Cells were treated with JAKi/DMSO (5 μM) for 1 h and stimulated with OSM (10 ng/ml) for 48 h. Non-migrating cells were removed from the upper surface by gentle scrubbing. Migrating cells attached to the lower membrane were fixed with 4% paraformaldehyde and stained with 0.1% crystal violet. Cells from five random high-power fields for each well were counted to assess the average number of invading cells.

### MMP 3-Plex MSD Assay

Supernatants from stimulated PsAFLS (2x10^4^ cells/well) seeded in 96-well plates were harvested for MMP1, MMP3 and MMP9 analysis by MSD assay (Meso Scale Diagnostics, USA) and MMP expression was measured according to manufacturer’s protocol.

### Statistical Analysis

Statistical analyses were performed using Prism 8 software. Wilcoxon Signed Rank test, one-way analysis of variance (ANOVA), Friedman Test with Dunn’s multiple comparison were utilised. *p* values of less than 0.05 (*p < 0.05), 0.01 (**p < 0.01), 0.001 (***p < 0.001) and 0.0001 (****p < 0.0001) were determined as statistically significant. All raw data are available on request.

## Results

### JAK Inhibitors Alter PsAFLS Secretion of Pro-Inflammatory Mediators Induced by OSM

**Figure 1:**
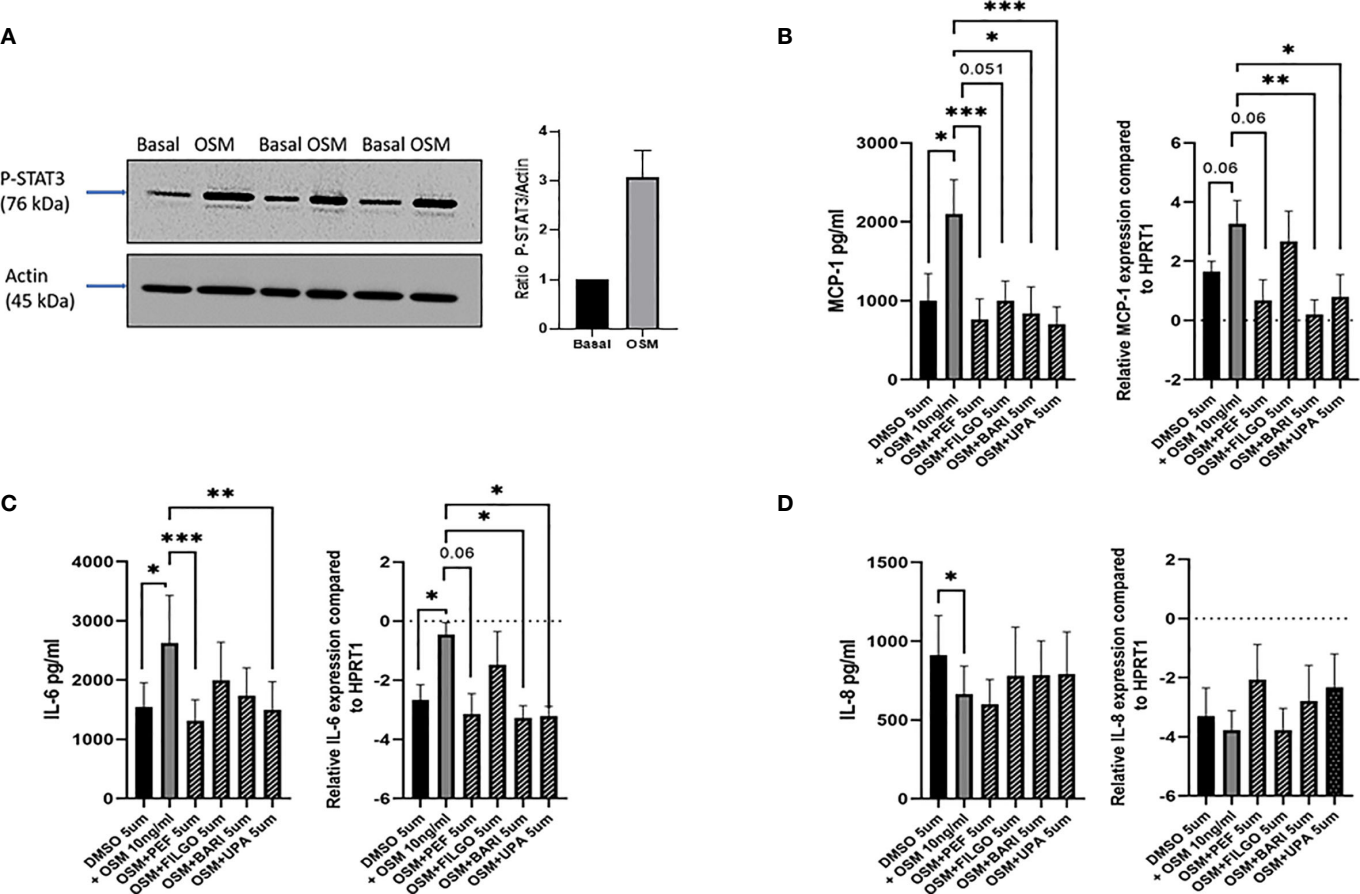
Effect of JAKi on the OSM driven expression of pro-inflammatory mediators in PsAFLS. **(A)** Representative western blot images and bar graph showing pSTAT3 expression in PsAFLS stimulated with/without OSM for 24 h. **(B–D)** PsAFLS were treated with JAKi (5 µM) or DMSO (5 µM) for 1 h and stimulated with OSM (10 ng/ml) for 24 h. Bar graphs demonstrating secretion by ELISA (n=7) and gene expression by real-time PCR (n=4-5) for MCP-1 **(B)** IL-6 **(C)** and IL-8 **(D)**. Values expressed ± SEM. *p < 0.05, **p < 0.01, ***p < 0.001 significantly different from DMSO+OSM control.

As OSM was utilised to active the JAK-STAT pathway, initial experiments assessed the effect of OSM on pSTAT3 expression. **Figure 1A** demonstrates that OSM stimulates pSTAT3 in n=3 separate PsAFLS. To assess the impact of JAKi, we initially determined their effect on a range of pro-inflammatory mediators. Firstly, we stimulated the PsAFLS with OSM and found that MCP-1 and IL-6 secretion (both p < 0.05) were significantly increased following stimulation compared to control (**Figures 1B, C**). IL-6 gene expression was also significantly increased (p < 0.05), with an increasing trend observed for MCP-1 gene expression (**Figures 1B, C**). MCP-1 secretion was significantly reduced by Peficitinib, Upadacitinib (both p < 0.001) and Baricitinib (p < 0.05) (**Figure 1B**). Although not significant, Filgotinib also showed a strong decrease in MCP-1 secretion. In parallel, inhibition was also observed at gene level, with Baricitinib (p < 0.01) and Upadacitinib (p < 0.05) significantly decreasing MCP-1 mRNA expression (**Figure 1B**). Similarly, JAKi also reduced OSM-induced expression of IL-6 at both the protein and gene level (**Figure 1C**). Peficitinib (p < 0.001) and Upadacitinib (p < 0.01) displayed significant inhibition of IL-6 (**Figure 1C**). This observation was also observed at gene level with Baricitinib (p < 0.05) and Upadacitinib (p < 0.05) showing significant reductions in IL-6 expression. Although not significant, Peficitinib also displayed an inhibitory capacity on IL-6 gene expression (**Figure 1C**). In contrast to both MCP-1 and IL-6 expression, OSM significantly reduced IL-8 expression (p < 0.05) compared to control (**Figure 1D** **)**. JAKi showed no significant effect on IL-8 secretion, however, there was an increasing trend observed for IL-8 mRNA expression (**Figure 1D**).

### JAK Inhibitors Reduce the OSM-Induced Shift to Glycolysis in PsAFLS

**Figure 2:**
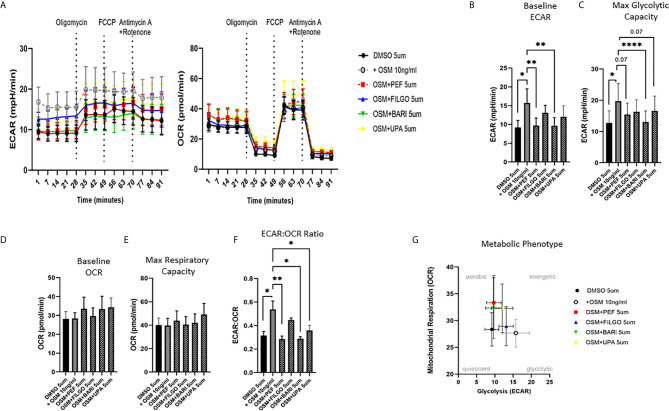
JAKi reduce the increased glycolytic profile of PsAFLS induced by OSM. **(A)** PsAFLS were treated with JAKi (5 µM) or DMSO (5 µM) for 1 h and stimulated with OSM (10 ng/ml) for 24 h. Average seahorse profiles demonstrating extracellular acidification rate (ECAR) (glycolysis) and oxygen consumption rate (OCR) (oxidative phosphorylation). **(B)** Representative bar graphs demonstrating baseline ECAR, **(C)** maximal glycolytic capacity, **(D)** baseline OCR, **(E)** maximal respiratory capacity, and **(F)** ECAR : OCR ratio. **(G)** Metabolic phenotype profile of PsAFLS, n=6. Values expressed as mean +/- SEM, *p < 0.05, **p < 0.01, ****p < 0.0001 significantly different from DMSO+OSM control.

**Figure 3:**
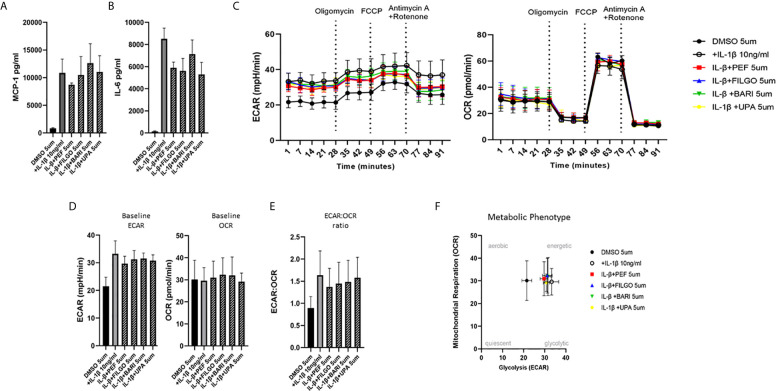
Effect of JAKi on the IL-1β-induced expression of pro-inflammatory mediators and bioenergetic profile in PsAFLS. **(A, B)** PsAFLS were treated with JAKi (5 µM) or DMSO (5 µM) for 1 h and stimulated with IL-1β (10 ng/ml) for 24 h. Bar graphs demonstrating secretion of **(A)** MCP-1 and **(B)** IL-6 by ELISA (n = 3). **(C)** Average seahorse profiles demonstrating extracellular acidification rate (ECAR) (glycolysis) and oxygen consumption rate (OCR) (oxidative phosphorylation). **(D)** Representative bar graphs demonstrating baseline ECAR and OCR and **(E)** ECAR : OCR ratio. **(F)** Metabolic phenotype profile in PsAFLS, n = 3. Values expressed as mean +/- SEM.

**Figure 4:**
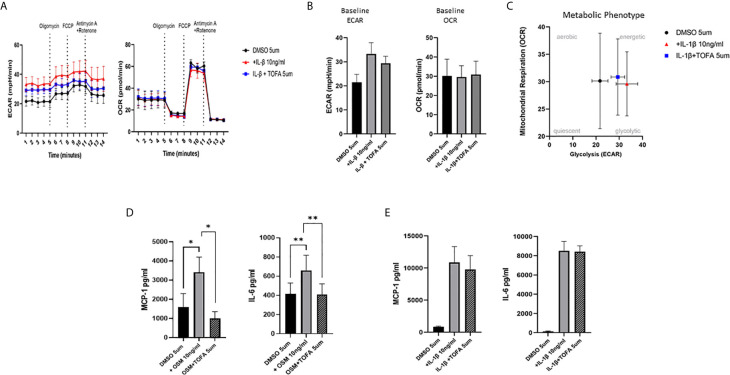
Effect of Tofacitinib on the IL-1B or OSM induced expression of pro-inflammatory mediators and bioenergetic profile in PsAFLS. **(A)** Average seahorse profiles demonstrating extracellular acidification rate (ECAR) (glycolysis) and oxygen consumption rate (OCR) (oxidative phosphorylation). **(B)** Representative bar graphs demonstrating baseline ECAR and OCR. **(C)** Metabolic phenotype profile of PsAFLS, n=3. **(D, E)** PsAFLS were treated with Tofacitinib (5 µM) or DMSO (5 µM) for 1 h and stimulated with OSM (10ng/ml) (n=7) or IL-1β (10 ng/ml) (n=3) for 24 h. and MCP-1 and IL-6 quantified. Values expressed as mean +/- SEM *p < 0.05, **p < 0.01.

To examine whether the inhibitory effect of JAKi on pro-inflammatory mediators is paralleled by a shift in metabolism, we analysed the two major energy pathways: oxidative phosphorylation (OCR) and glycolysis (ECAR) in real time, using the Seahorse XFe- Analyser. **Figure 2A** displays the average bioenergetic profiles for ECAR and OCR of PsAFLS before and after injections of mitochondrial inhibitors: oligomycin, carbonyl cyanide-p-trifluoromethoxyphenylhydrazone (FCCP), antimycin A and rotenone in the presence of OSM. As shown in the seahorse profiles, stimulation with OSM increased the ECAR with no effect observed for OCR (**Figure 2A**). Quantification demonstrated a significant increase in baseline ECAR (p < 0.05) (**Figure 2B**) with no effect observed for baseline OCR (**Figure 2D**). Similarly, max glycolytic capacity (p < 0.05), but not max respiratory capacity was significantly increased by OSM stimulation (**Figures 2C, E**). Treatment with JAKi demonstrated a decrease in the ECAR bioenergetic profile for all JAKi, with minimal change observed for the OCR bioenergetic profile (**Figure 2A**). All JAKi reduced the OSM- induced glycolytic capacity of PsAFLS with a significant decrease in basal glycolytic capacity in response to Peficitinib (p < 0.01) and Baricitinib (p < 0.01) (**Figure 2B**), and a significant decrease in Maximal Glycolytic Capacity in response to Baricitinib (p < 0.0001) (**Figure 2C**). No change in basal respiration (**Figure 2D**), or maximal respiratory capacity in response to JAKi was observed (**Figure 2E**). This therefore resulted in a significant decrease in the ECAR : OCR ratio, signifying a shift away from glycolytic mechanisms and towards a reliance on mitochondrial respiration. This was most significant following treatment with Peficitinib (p < 0.01), Baricitinib (p < 0.05) and Upadacitinib (p < 0.05) (**Figure 2F**). Filgotinib also decreased both the glycolytic capacity and ECAR : OCR ratio, but this did not reach significance (**Figure 2F**). The impact of JAKi on PsAFLS bioenergetics is clearly demonstrated in the overall metabolic profile, whereby the glycolytic profiles of PsAFLS induced by OSM, shift towards a more quiescent state following treatment with JAKi (**Figure 2G**). To examine if JAKi alter secondary downstream effects of other cytokines that do not signal through the JAK-STAT pathway, we examined their effect on IL-1β-induced PsAFLS (**Figures 3** and **4**). JAKi have no effect on IL-1β-induced MCP-1 secretion (**Figure 3A**). While some inhibitory effect was observed for IL-1β-induced IL-6 expression, this is not significant (**Figure 3B**). We also examined the bioenergetic profile of PsAFLS stimulated with IL-1β and the four JAKi (**Figures 3C–F**), in addition to Tofacitinib (**Figures 4A–C**). IL-1β increased the ECAR but not the OCR of the cells, with the JAKi’s including Tofacitinib showing minimal effect on either energetic pathways (**Figures 3C–F** and **Figures 4A–C**). Tofacitinib which predominantly inhibits JAK3 and to a lesser extent JAK2 significantly inhibited both MCP-1 and IL-6 in response to OSM (**Figure 4D**). Similar to the other JAKi, Tofacitinib had no effect on IL-1β-induced MCP-1 and IL-6 secretion (**Figure 4E**).

### OSM Driven PsAFLS Invasion Is Inhibited by JAKi

**Figure 5:**
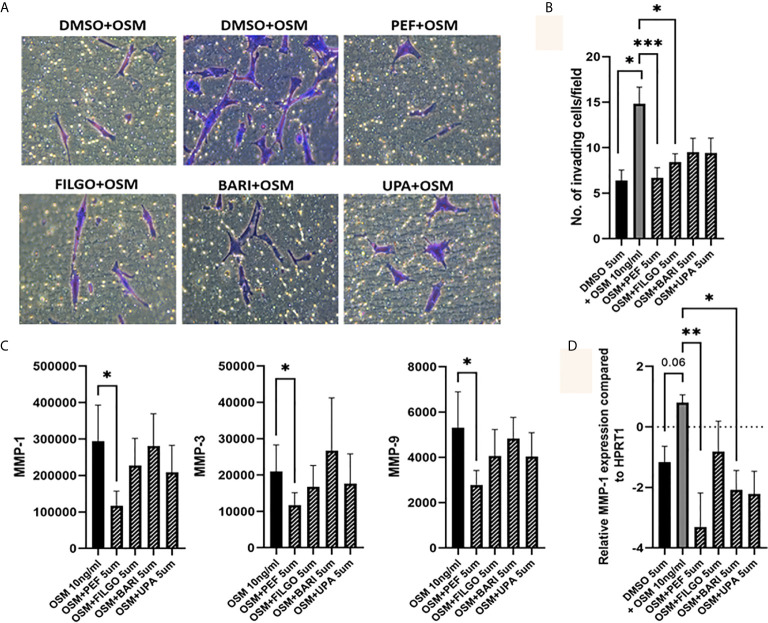
OSM driven invasion by PsAFLS is blocked by JAKi. **(A)** Representative photomicrographs and **(B)** bar graph showing PsAFLS invasion following treatment with JAKi (5 µM) or DMSO (5 µM) for 1 hr and stimulated with OSM (10 ng/ml) for 24 h, n=6 (magnification X20). **(C)** Representative bar graph showing MMP-1, MMP-3 and MMP-9 expression by MSD ELISA (n=7) and **(D)** gene expression analysis of MMP-1 by real-time PCR (n=5). Values expressed as the mean +/- SEM, *p < 0.05, **p < 0.01, ***p < 0.001 significantly different from DMSO+OSM control.

To further examine the effect of JAKi on PsAFLS pathogenic function, we examined the effect of JAKi on the invasive capacity of PsAFLS using Transwell Matrigel™ invasion chambers following stimulation with OSM. Representative images of PsAFLS invasion in unstimulated cells, OSM stimulated cells and OSM stimulated cells following treatment with JAKi are shown in **Figure 5A**. Quantitative analysis demonstrated the significant increase in invasive capacity following OSM stimulation (p < 0.05) compared to unstimulated cells (**Figure 5B**). This was significantly impacted by treatment with all JAKi, however Peficitinib (p < 0.001) and Filgotinib (p < 0.05) had the most significant reductions (**Figure 5B**). In parallel, we examined the effect of JAKi on the secretion of the cartilage destructive matrix metalloproteinase enzymes. Peficitinib significantly decreased MMP-1 (p < 0.05), MMP-3 (p < 0.05) and MMP-9 (p < 0.05). While a decrease for all three MMPs was also observed in Upadacitinib, Filgotinib and Baricitinib treated cells, this did not reach significance (**Figure 5C**). In response to the OSM induced MMP-1 expression, gene analysis showed similar decreasing trends, with Peficitinib (p < 0.01) and Baricitinib (p < 0.05) displaying the greatest inhibition. Although not significant, Filgotinib and Upadacitinib both reduced MMP-1 expression (**Figure 5D**).

### JAK Inhibitors Block PsAFLS Migration Promoted by OSM

**Figure 6:**
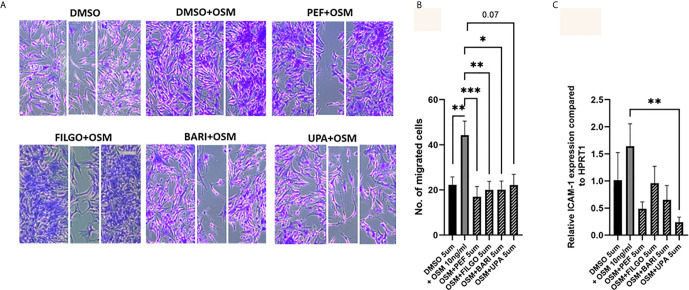
JAKi inhibit OSM induced migration of PsAFLS. **(A)** Representative photomicrographs and **(B)** bar graph showing PsAFLS migration following treatment with JAKi (5 µM) or DMSO (5 µM) for 1 hr and stimulated with OSM (10 ng/ml) for 24 h, n=8 (magnification X10). **(C)** Gene expression analysis of ICAM by real-time PCR (n=5). Values expressed as mean +/- SEM. *p < 0.05, **p < 0.01, ***p < 0.001 significantly different from DMSO+OSM control.

Finally, the capacity of PsAFLS to migrate within the joint environment is associated with progressive and destructive joint disease, therefore, we next investigated the role of JAKi on migration of PsAFLS using a wound repair scratch assay. **Figure 6A** shows representative images demonstrating the increased migratory capacity of PsAFLS in response to OSM compared to unstimulated cells, in addition to the inhibitory effect of JAKi on PsAFLS migration, where repopulation of wound margins was inhibited by JAKi. Quantitative analysis demonstrated that OSM significantly induced migration of PsAFLS (p < 0.01) compared to basal control (**Figure 6B**). However, Peficitinib (p < 0.001), Filgotinib (p < 0.01) and Baricitinib (p < 0.05) all significantly decreased PsAFLS migration across the wound margins (**Figure 6B**). Although not significant, Upadacitinib also demonstrated strong inhibition of PsAFLS migration (**Figure 6B**). As migration of FLS is aided by adhesion molecules we also determined if JAKi influenced ICAM expression. Gene analysis showed a significant inhibition of OSM- induced ICAM expression by Upadacitinib (p < 0.01), all other inhibitors displayed decreases in ICAM expression, although these did not reach significance (**Figure 6C**).

A summary table outlining the main effects of each JAKi can be found in **Table 2**.

**Table 2:** Summary of JAKi functions.

JAKi	Target	IL-6	MCP-1	ECAR/OCR Ratio	Invasion	MMPs	ICAM-1	Migration
**Peficitinib**	Pan JAKi	↓↓↓	↓↓↓	↓↓	↓↓↓	↓	↓	↓↓↓
**Filgotinib**	JAK 1	-	-	-	↓	-	↓	↓↓
**Baricitinib**	JAK1/2	↓	↓	↓	-	↓	↓	↓
**Upadacitinib**	JAK1	↓↓↓	↓↓	↓	-	-	↓↓	-

## Discussion

In this study, we identified the impact of four JAKi inhibitors; Peficitinib, Filgotinib, Baricitinib and Upadacitinib on the pathogenic phenotype observed in PsAFLS. JAKi are an encouraging class of drugs for the treatment of PsA, as evidence of increased JAK/STAT signalling has been shown at the site of inflammation (15, 21). To date, no study has examined the role of these JAKi on primary cells isolated from PsA synovial tissue. We utilised OSM as a stimulant, as it signals through the JAK-STAT pathway, specifically activating JAK1, JAK2 and to a lesser degree TYK2 (25). OSM is increased at the site of inflammation, in addition, several studies have shown that OSM drives synovial fibroblast invasive mechanisms (31–35). Using OSM to drive this inflammatory response, we demonstrate that JAKi significantly decreased the secretion of key pro-inflammatory mediators; MCP-1 and IL-6. This was accompanied by changes in the bioenergetics of the cells, whereby JAKi decreased the glycolytic profile of the PsAFLS resulting in a shift towards a more oxidative phosphorylated/quiescent phenotype. Finally, we demonstrated the ability of JAKi to inhibit the pathogenic function of PsAFLS by significantly decreasing their invasive and migratory capacity. While all JAKi inhibitors decreased pro-inflammatory and metabolic mechanisms in PsAFLS, this was most pronounced for Peficitinib. These data demonstrate that JAK/STAT signalling mediates pro-inflammatory mechanisms that drive PsA pathogenesis, an effect inhibited with the use of JAKi.

In this study, we show that JAKi significantly reduce the secretion of OSM-induced pro-inflammatory mediators MCP-1 and IL-6, while displaying an increasing trend in IL-8 secretion, this differential regulation is consistent with the pleiotropic effects of OSM (36). We also show that Peficitinib, Baricitinib and Upadacitinib displayed the most significant inhibition of MCP-1 and IL-6 secretion, while Filgotinib also decreased cytokine secretion, this did not reach significance. The role for JAK-STAT signalling in PsA is consistent with studies showing increased expression of pSTAT3 and pSTAT1 in PsAFLS and PsA synovial tissue (21). Studies in psoriasis have shown an increase in pSTAT expression localised to the epidermal hyper-proliferation layer (37, 38). Furthermore, the effect of JAKi is consistent with previous reports showing Tofacitinib, Peficitinib and Baricitinib inhibit IL-6 and MCP-1 expression in RAFLS (39, 40). As these JAKi can inhibit multiple pro-inflammatory mediators simultaneously and rescue function, they may act as a superior treatment for PsA compared to blockade of one specific cytokine.

To examine if the effect of JAKi on pro-inflammatory function also alters the energy profile of PsAFLS, we investigated the two major energy pathways, glycolysis and oxidative phosphorylation using real-time Seahorse Technology. Changes in metabolism have been observed at the site of inflammation in both PsA and RA, due most likely to environmental factors within the joint resulting in a hypoxic microenvironment (41–44). Previous studies have shown a shift to a more glycolytic profile in RAFLS compared to OAFLS (45), with several studies demonstrating elevated levels of metabolic intermediates and increased activity of key glycolytic enzymes in both the RA and PsA synovium/cells (46–48). However, this is the first study to compare the bioenergetics of PsAFLS in response to treatment with JAKi. We show that JAKi reduced the glycolytic shift in favour of a more oxidative state, similar to a cell in quiescence. These findings are consistent with a report by McGarry et al. using Tofacitinib which inhibited glycolysis along with key glycolytic genes *HK2, GSK3A, PDK1* and *HIF1α* in RAFLS (39). Although all JAKi displayed inhibition, Peficitinib and Baricitinib significantly reduced the rates of glycolysis, with Peficitinib having the greatest effect on the ECAR/OCR ratio. Regulation of the metabolic pathways has been strongly linked with resolution of inflammation in the inflamed joint, with several studies demonstrating that metabolic blockade inhibits inflammation *in vitro*, *ex vivo* and *in vivo* models of arthritis (39, 42, 44, 45, 47–51).

Interestingly, interactions between JAK-STAT signalling and metabolic pathways have been demonstrated in previous studies. Blockade of the key glycolytic enzyme PFKFB3 inhibits pSTAT3 activation in RAFLS (41, 51, 52). In turn, STAT3 itself can regulate glycolysis through HK2 in cancer cells (41, 51, 52), and plays a key regulatory role in mediating interactions between HIF1α and PKM2 (41, 51–54). Interplay between STAT3 and Sirtuin-1 has also been demonstrated to regulate oxygen consumption, ETC complex activity and metabolic intermediates in the mitochondria (53, 54). Indeed, studies have suggested that this effect may be due to localised STAT3 expression in the mitochondria which modulates the activity of complex I and II (52), however, other studies suggest alternative mechanisms, either *via* additional transcriptional regulation, or indirect activation of mitochondrial signalling pathways (55). In context of the inflamed joint, STAT3 interacts with various other key signalling pathways including HIF1a, Notch and NFκB all of which regulate each other’s activation through complex positive and negative feedback loops in the PsA/RA joint (56). Therefore, the use of JAKi in metabolically reprogramming these cells may aid in reducing their inflammatory aggressive phenotype in PsA.

We also determined the effect of JAKi on PsAFLS function by examining their invasive and migratory capacity. All JAKi showed a striking inhibition of invasion by PsAFLS, although Peficitinib and Filgotinib displayed the strongest effect. In parallel, all JAKi significantly inhibited PsAFLS migration. While the precise mechanism by which JAKi impacts invasion and migration is unclear, Peficitinib significantly reduced MMP-1, MMP-3, and MMP-9 secretion, while the other JAKi showed a slight decrease. Consistent with our data, several other studies using RAFLS have reported inhibition of MMP-1 and MMP-3 by both Tofacitinib and Peficitinib (29, 39, 40), in addition to the inhibitory effect of Tofacitinib on PsAFLS invasion, network migration and migration (21). Furthermore, in PsA synovial explants, Tofacitinib inhibits MMP-3 expression and the overall MMP-3/TIMP ratio (21), thus reducing the ability of FLS to invade the tissue thereby reducing joint destruction. Other pathways involved in FLS invasion and migration include Integrin-cytoskeletal pathways that bridge cell–cell and cell–ECM interactions (57), with previous studies showing that JAK-STAT signalling regulates RA-FLS lamellipodia formation and RhoGTPases, key proteins involved in cellular movement (58). As migration of these cells to the joint is aided by adhesion molecules, we also show that ICAM expression is strongly reduced following treatment with JAKi. Similar effects were seen with RANKL, where Peficitinib and Tofacitinib decreased expression in RAFLS (34). Other potential mechanisms include the YAP pathway which has been implicated in RAFLS invasiveness (59, 60). Indeed, studies in fibroblasts from other disease settings have shown complex interactions between metabolic pathways and YAP/TAZ signalling (61). Therefore, the use of JAKi in PsA may help in reducing inflammation induced by infiltrating FLS to the inflamed joint.

Finally, in this study we utilised OSM as an activator of the JAK/STAT pathway, however, it is only one of many cytokines implicated in PsA pathogenesis that acts through this pathway, including IL-12, IL-23, IL-22 and IFNγ. Thus, there are limitations to the interpretation. While outside the scope of this study, an ideal model would be to culture PsAFLS with a cocktail of all the relevant cytokines that are known to be increased in the PsA joint in the presence or absence of JAKi.

In conclusion, this study demonstrates the effect of JAKi in targeting PsAFLS function *in vitro*, inhibiting invasive, migration and metabolic mechanisms leading to resolution of inflammation. These findings support the role for JAKi in patients with inadequate responses to current PsA therapies.

## Data Availability Statement

The raw data supporting the conclusions of this article will be made available by the authors, without undue reservation.

## Ethics Statement

The studies involving human participants were reviewed and approved by St Vincent’s University Hospital Research Ethics Committee. The patients/participants provided their written informed consent to participate in this study.

## Author Contributions

AO’B, MH, VM, SW, and KF performed the experiments, analysed the data, and prepared the manuscript. KF also processed clinical samples. UF conceived the experimental approach, analysed the data, and supervised and prepared the manuscript. DV conceived the experimental approach, collected the clinical samples, analysed the data, supervised the study, and prepared the manuscript. All authors contributed to the article and approved the submitted version.

## Funding

This work was supported by the CARD Charity and Arthritis Ireland.

## Conflict of Interest

The authors declare that the research was conducted in the absence of any commercial or financial relationships that could be construed as a potential conflict of interest.
